# A Chloroplast-Localized Glucose-6-Phosphate Dehydrogenase Positively Regulates Stripe Rust Resistance in Wheat

**DOI:** 10.3390/ijms24010459

**Published:** 2022-12-27

**Authors:** Xiaobo Wei, Xueling Huang, Weiling Yang, Xinran Wang, Tao Guan, Zhensheng Kang, Jie Liu

**Affiliations:** 1 State Key Laboratory of Crop Stress Biology for Arid Areas, Northwest A&F University, Yangling 712100, China; 2 College of Life Sciences, Northwest A&F University, Yangling 712100, China; 3 College of Plant Protection, Northwest A&F University, Yangling 712100, China

**Keywords:** glucose-6-phosphate dehydrogenase, wheat stripe rust, chloroplast, *Puccinia striiformis* f. sp. *tritici*, reactive oxygen species

## Abstract

Glucose-6-phosphate dehydrogenase (G6PDH), the rate-limiting enzyme of the pentose phosphate pathway (PPP), plays a pivotal role in plant stress responses. However, the function and mechanism of G6PDHs in crop plants challenged by fungal pathogens remain poorly understood. In this study, a wheat G6DPH gene responding to infection by *Puccinia striiformis* f. sp. *tritici* (*Pst*), designated *TaG6PDH2*, was cloned and functionally identified. *TaG6PDH2* expression was significantly upregulated in wheat leaves inoculated with *Pst* or treated with abiotic stress factors. Heterologous mutant complementation and enzymatic properties indicate that *TaG6PDH2* encodes a G6PDH protein. The transient expression of *TaG6PDH2* in *Nicotiana benthamiana* leaves and wheat protoplasts revealed that TaG6PDH2 is a chloroplast-targeting protein. Silencing *TaG6PDH2* via the barley stripe mosaic virus (BSMV)-induced gene silencing (VIGS) system led to compromised wheat resistance to the *Pst* avirulent pathotype CYR23, which is implicated in weakened H_2_O_2_ accumulation and cell death. In addition, TaG6PDH2 was confirmed to interact with the wheat glutaredoxin TaGrxS4. These results demonstrate that *TaG6PDH2* endows wheat with increased resistance to stripe rust by regulating reactive oxygen species (ROS) production.

## 1. Introduction

The pentose phosphate pathway (PPP) is a critical carbohydrate metabolic pathway. PPP, which exists in all eukaryotes and prokaryotes except for Archaea [1], is necessary for providing reducing power and intermediary metabolites in the plant life cycle. Nicotinamide adenine dinucleotide phosphate (NADPH), a reducing energy equivalent, is primarily generated via the PPP under dark conditions or in non-synthetic tissues and is used in reductive biosynthesis to maintain the cellular redox state [2]. Meanwhile, NADPH is the substrate for the NADPH oxidases that catalyze O_2_ to form O_2_^−^ outside of plant cells, which leads to reactive oxygen species (ROS) accumulation. Although ROS are thought to play important roles in varied physiological and molecular processes, an excess of ROS can provoke oxidative damage, ultimately inducing cell death. Thus, the fine-tuned regulation of ROS homeostasis is required for normal plant growth, development, and stress responses [3]. In general, glucose-6-phosphate dehydrogenase (G6PDH) is considered a main regulator of the PPP and the key control point for NADPH production [4]. G6PDH not only facilitates ROS accumulation via the oxidase-dependent NADPH route but can also provide NADPH to the antioxidant system to scavenge ROS, which is critical for maintaining cellular redox homeostasis.

G6PDH catalyzes the conversion of glucose-6-phosphate (G6P) to 6-phosphogluconate (6PG), produces NADPH, and is the initial and rate-limiting enzyme in the PPP [5]. Based on varying subcellular localizations, plant G6PDHs are classified into two types of isoforms: cytosolic (Cy-G6PDHs) and plastidic (P-G6PDHs), the regulation of which is controlled by discrete mechanisms [6]. According to gene expression patterns and enzymatic characteristics [7,8], P-G6PDHs are further divided into plastidic isoforms 1 (P1-G6PDHs) and plastidic isoforms 2 (P2-G6PDHs). Cy-G6PDHs, lacking transit peptides at the N-terminals of amino acid sequences, generally exhibit extremely low sensitivity to NADPH. The activity of plastidic enzymes is subject to feedback inhibition by NADPH and redox regulation, which adjusts the adaptation of PPP under light conditions [9,10]. P1-G6PDHs are mainly expressed in green tissues, which are highly restricted by NADPH and tightly regulated by light to provide effective photosynthesis. P2-G6PDHs are mostly detected in roots and heterotrophic tissues and show less sensitivity to NADPH inhibition than P1-G6PDH [11,12]. The classification of G6PDHs implies that each isoform may carry out different functions in the plant life cycle.

Numerous studies have demonstrated that G6PDHs play pivotal roles in plant stress responses. For example, transgenic *Arabidopsis* plants overexpressing wheat *TaG6PDH* displayed greater ROS-scavenging abilities and elevated tolerance to cold stress compared with wild-type plants [13]. Similarly, the overexpression of the NaCl-induced soybean Cy-G6PDH isoform *GmG6PD7* resulted in enhanced tolerance to salt stress by decreasing ROS accumulation in Arabidopsis [14]. Huang et al. also found that G6PDHs are narrowly concerned with NADPH oxidase-mediated ROS production and hypersensitive response (HR)-associated cell death under aluminum (Al) stress. Increasing G6PDH activity can promote ROS accumulation and cell death, whereas the inhibition of G6PDH activity has the opposite effect [15]. G6PDHs have also been shown to contribute to plant pathogen resistance. For instance, the silencing of a P2-type G6PDH isoform led to reduced ROS production and cell death in tobacco invaded by pathogens [16]. Wei et al. found that G6PDHs were deployed to counter *Penicillium expansum* by regulating ROS metabolism and NADPH production [17]. In addition, G6PDH activity is correlated with host resistance to virus multiplication [18]. Taken together, these results suggest that G6PDHs contribute to plant stress tolerance by interfering with the ROS balance. 

Wheat (*Triticum aestivum*. L) is often cultivated under unfavorable environmental conditions and subjected to various stresses that pose a tremendous threat to food security. Stripe rust, caused by *Puccinia striiformis* f. sp. *tritici* (*Pst*), results in severe yield losses [19]. During *Pst* infection, ROS burst occurs to protect the wheat against fungal invasion, while *Pst* secretes antioxidant enzymes, PsSODs, and PsCAT1 as virulence factors to promote *Pst* infection by directly scavenging host-derived ROS [20,21,22]. Thus, ROS production is a decisive point at which to determine the outcome of wheat–rust interactions. Previous research found a *G6PDH* gene in wheat, designated *TaG6PDH2*, that is significantly induced in wheat–*Pst* interactions [23]. However, the function and mechanism of *TaG6PDH2* remain unknown. In the current study, the transcription abundance of *TaG6PDH2* was increased in wheat leaves infected by *Pst* and treated with abiotic stress factors. The subcellular localization and heterologous expression indicated that TaG6PDH2 is a G6PDH that is located in the chloroplast. Knocking down *TaG6PDH2* via a virus-induced gene silencing (VIGS) system reduced the resistance of wheat to *Pst*. In addition, TaG6PDH2 interacted with the wheat glutaredoxin TaGrxS4. These results shed light on the contributions of *TaG6PDH2* to wheat resistance against stripe rust. TaG6PDH2 may function as a positive regulator in defense responses by increasing the accumulation of ROS during the early *Pst* infection stage.

## 2. Results

### 2.1. Cloning and Sequence Analysis of Wheat G6PDH 

Based on the transcriptome analysis of *Pst*-infected wheat leaves, a *G6PDH* gene was found to be significantly upregulated in the wheat–*Pst* interaction. The BLAST results indicated that three subgenome copies exist in chromosomes 2A, 2B, and 2D. The phylogenetic relationship of *G6PDHs* from wheat and *A. thaliana* showed that the induced G6PDH was clustered with AtG6PD2 and AtG6PD3 (Supplementary Figure S1), demonstrating that it belonged to the P2-type G6PDH. Accordingly, it was designated as *TaG6PDH*2 due to its higher sequence similarity with AtG6PD2. The encoding sequences of the three subgenome copies of *TaG6PDH2* exhibited a sequence identity of 95.1% (Supplementary Figure S2), and the deduced amino acid sequences of these three alleles share 96.7% sequence similarity (Supplementary Figure S3). In addition, the core domains G6PD_N (Pfam ID PF00479) and G6PD_C (Pfam ID PF02781) were highly conserved (Supplementary Figure S3). These data suggest that these three subgenome copies may have the same biological functions. Thus, *TaG6PDH2-2D* was used for further analysis in this study. 

The full-length coding sequence (CDS) of *TaG6PDH2* was cloned from *Pst*-infected wheat leaves using RT-PCR. A sequence analysis revealed that *TaG6PDH2* consists of 1779 nucleotides that are predicted to encode a polypeptide of 592 amino acids (aa) with a calculated molecular weight of 65.8 kDa and an isoelectric point (pI) of 8.39. Subcellular localization predicted TaG6PDH2 to have a 38 aa chloroplast transit peptide (cTP) at the N-terminal localized in the chloroplast (Supplementary Figure S3A). The phylogenetic tree of TaG6PDH2 was constructed with homologous proteins from various species, indicating that TaG6PDH2 is closely related to the G6PDHs from *Triticum urartu*, *Aegilops tauschii* subsp. *tauschii,* and *Hordeum vulgare* (Supplementary Figure S4).

### 2.2. TaG6PDH2 Is Localized in the Chloroplast

**Figure 1:**
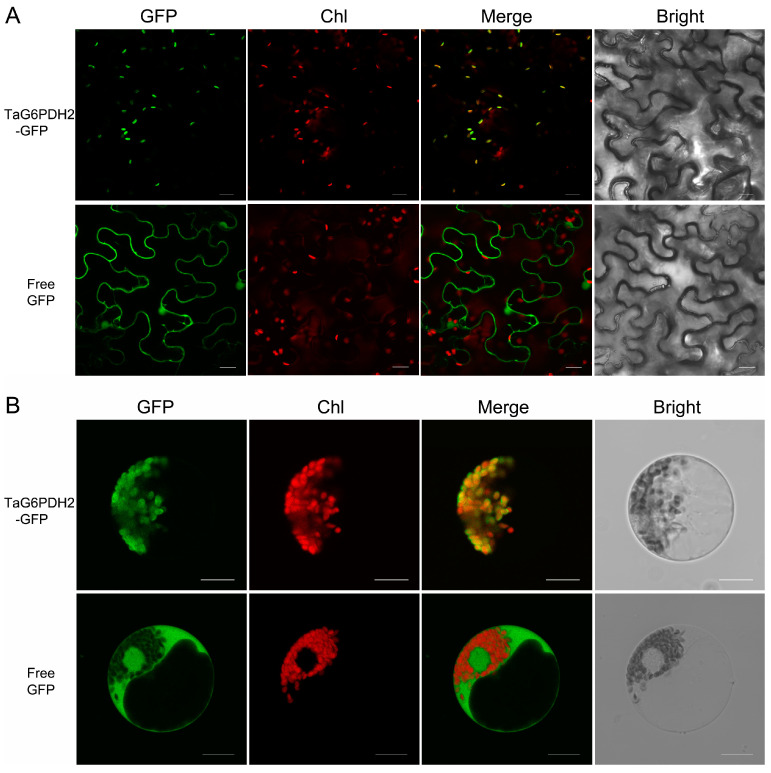
Subcellular localization of TaG6PDH2 in plants. (**A**) Transient expression of free GFP or TaG6PDH2-GFP fusion protein in *N. benthamianain* leaves. (**B**) Free GFP or TaG6PDH2-GFP fusion proteins were expressed in wheat protoplasts. All signals were detected by an Olympus FV3000 confocal microscope. Scale bars, 20 μm. Chl, chlorophyll. GFP fluorescence is shown in green. Red fluorescence represents chlorophyll auto-fluorescence. Bright-field images indicate that the identical field was observed under white light. Merged GFP and chlorophyll images are presented.

P2-type G6PDHs are chloroplast-targeting proteins [7,8,16,24]. To illustrate the subcellular localization of TaG6PDH2, the TaG6PDH2-GFP fusion protein and free GFP protein were transiently expressed in tobacco leaves and wheat protoplasts, respectively. Similar results were observed through a confocal laser scanning microscopy analysis. As expected, fluorescence signals from the free GFP were detected throughout the cytoplasm and nucleus, while the TaG6PDH2-GFP fusion protein was only found in the chloroplasts (Figure 1A,B). In addition, the TaG6PDH2 with cTP deletion failed to localize to the chloroplasts (Supplementary Figure S5). Consistent with our prediction of the subcellular localization of TaG6PDH2, these results suggest that TaG6PDH2 was localized to the chloroplasts.

### 2.3. TaG6PDH2 Is Induced by Pst, Hormone Elicitors, and Abiotic Stresses

**Figure 2:**
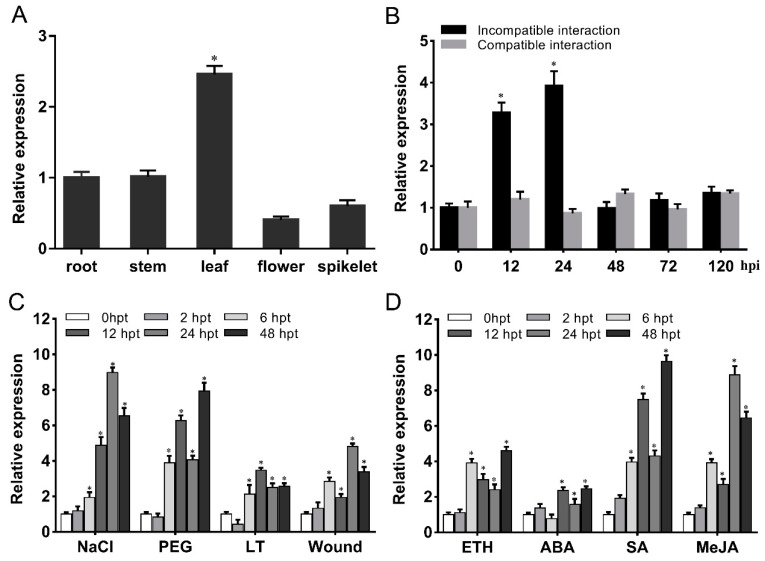
Expression patterns of *TaG6PDH2* under different treatments. (**A**) *TaG6PDH2* expression profiles in different wheat tissues. Samples were collected from roots, stems, leaves, flowers, and spikelets. Asterisks represent significant differences (*p* < 0.05) compared to the root, according to Student’s *t*-test. (**B**) Expression patterns of *TaG6PDH2* in *Pst-*infected wheat leaves. The wheat leaves were inoculated with *Pst* CYR23 or CYR31 and sampled at 0, 12, 24, 42, 72, and 120 hpi. Asterisks represent significant differences (*p* < 0.05) from 0 hpi, according to Student’s *t*-test. (**C**,**D**) *TaG6PDH2* expression patterns in response to abiotic stresses (**C**) and exogenous hormones (**D**). Samples were collected from treated wheat leaves at 0, 2, 6, 12, 24, and 48 hpt. Asterisks indicate significant differences (*p* < 0.05) compared to 0 hpt, according to Student’s t-test. PEG; polyethyleneglycol 6000; LT, low temperature; ETH, ethylene; ABA, abscisic acid; SA, salicylic acid; MeJA, methyl jasmonate.

The *TaG6PDH2* transcript levels in several wheat tissues were analyzed using qRT-PCR. *TaG6PDH2* was ubiquitously expressed, and the transcript abundance of *TaG6PDH2* in green leaves was more than that in roots, stems, flowers, and spikelets (Figure 2A). We conducted qRT-PCR to confirm the expression profile of *TaG6PDH2* during *Pst* infection. The expression of *TaG6PDH2* was upregulated at 12 and 24 h post-inoculation (hpi) in the wheat*–Pst* incompatible interaction, and the maximum transcript levels of *TaG6PDH2* were approximately 4-fold higher than in the control plants at 24 hpi. However, no significant change was observed in the wheat–*Pst* compatible interaction (Figure 2B). These results suggest that *TaG6PDH2* may have an important role in wheat resistance to *Pst* infection. *G6PDHs* are commonly implicated in responses to abiotic stresses [13,14,15]. To test whether *TaG6PDH2* participates against abiotic stresses in wheat, the transcript abundance of *TaG6PDH2* under different abiotic stresses, including cold, wound, drought, and salinity, was assayed. Polyethyleneglycol (PEG) 6000 treatment triggered a marked increase in the *TaG6PDH2* transcript abundance at 6 h post-treatment (hpt), reaching a peak at 48 hpt. The highest expression level of *TaG6PDH2* was about nine times higher than in the control plants at 24 hpt after NaCl treatment. A low-temperature (LT) treatment induced an upregulation of *TaG6PDH2* expression from 6 to 48 hpt. In addition, the transcript level of *TaG6PDH2* was significantly elevated under the wound treatment; the expression levels peaked at 24 hpt (Figure 2C). We also analyzed the *TaG6PDH2* transcript patterns in wheat leaves sprayed with salicylic acid (SA), ethylene (ETH), methyl jasmonate (MeJA), and abscisic acid (ABA). *TaG6PDH2* responded to treatments with these four exogenous hormones compared with the control plants, especially SA and MeJA (Figure 2D).

### 2.4. Functional Characterization of TaG6PDH2

**Figure 3:**
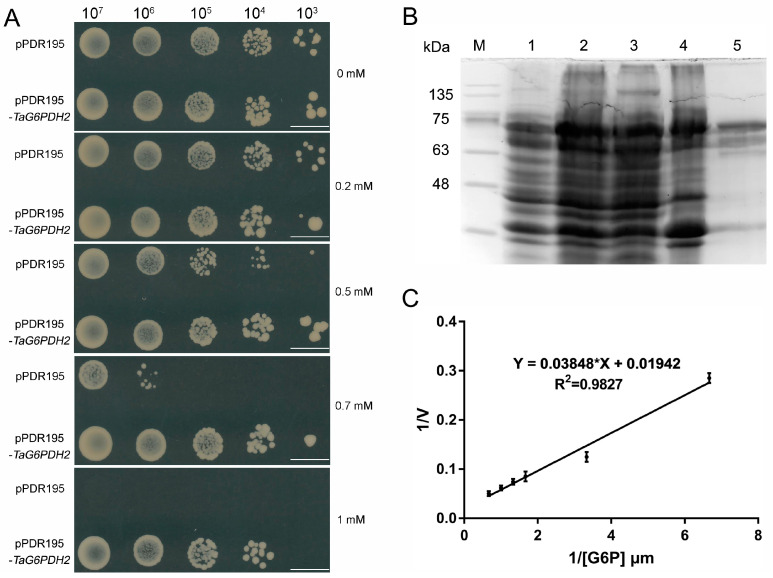
Heterologous expression of *TaG6PDH2* in *S. cerevisiae* and *E. coli*. (**A**) Functional analysis of *TaG6PDH2* in *S. cerevisiae* mutant strain YNL24C. Growth of YNL24C harboring either pDR195 or pDR195-*TaG6PDH2* on SD/−Ura plates containing different concentrations of H_2_O_2_. Scale bars, 1 cm. (**B**,**C**) Purification and enzymatic characterization of TaG6PDH2. (**B**) SDS-PAGE of samples at different purification stages of TaG6PDH2 in *E. coli* BL21 (DE3). Channel 1, control, uninduced *E. coli* cell lysates; channel 2, *E. coli* cell lysates induced by IPTG; channel 3, supernatant of bacterial lysate; channel 4, pellet of bacterial lysate; channel 5, purified fusion proteins; M, marker. (**C**) Linear regression equation presented by the double-reciprocal plot method (1/V vs. 1/[G6P]). Each point represents the average of three replicates with the standard error. * represents multiply.

To investigate the role of *TaG6PDH2*, the recombinant plasmid pDR195-*TaG6PDH2* was introduced into the *G6PDH*-deficient yeast mutant strain YNL241c, which exhibits enhanced sensitivity to H_2_O_2_ [25,26]. The positive transformants containing pDR195-*TaG6PDH2* were plated on an SD-Ura medium filled with several concentrations of H_2_O_2_. We found that the YNL241c mutants harboring pDR195-*TaG6PDH2* were more resistant to high concentrations of H_2_O_2_ than the control with the empty vector pDR195 (Figure 3A), indicating that TaG6PDH2 can partially rescue the tolerance of YNL241c to oxidative stress.

To identify the enzymatic characteristics of TaG6PDH2, the expression of the recombinant TaG6PDH2 protein was induced. The TaG6PDH2 proteins were further purified using a His-Trap FF affinity column. An intense band around 75 kDa, consistent with the predicted molecular weight of TaG6PDH2 plus the molecular weights of the Polyhistidine Affinity (6 × His) tag and the small ubiquitin-like modifier (sumo) tag, was detected using SDS-PAGE, indicating that the purified TaG6PDH2 protein was successfully obtained (Figure 3B). The *K*m of the purified TaG6PDH2 protein on the glucose-6-phosphate (G6P) substrate was calculated based on the linear regression equation presented using double-reciprocal plots. The kinetic parameters of TaG6PDH2 exhibited a *K*m_G6P_ of 1.98 mM (Figure 3C). In summary, TaG6PDH2 possessed the enzymatic activity of G6PDH.

### 2.5. Silencing of TaG6PDH2 Resulted in Reduced Wheat Resistance to Pst

**Figure 4:**
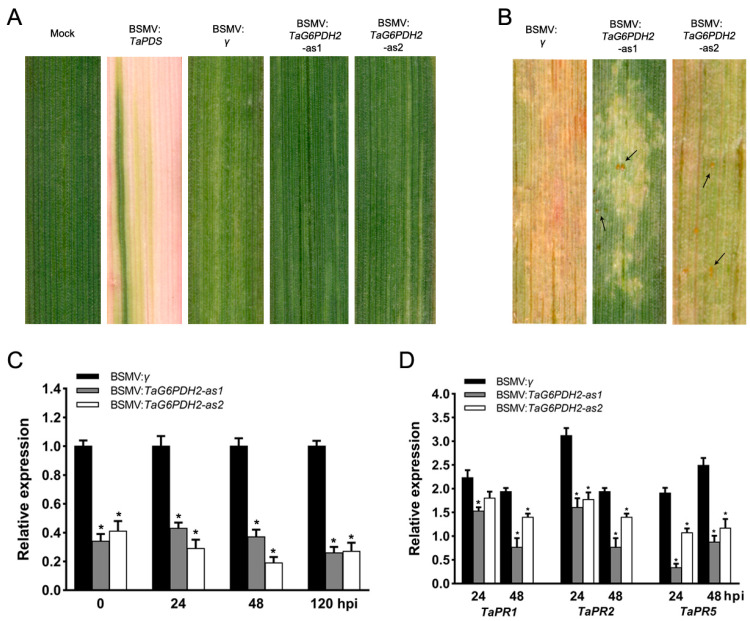
Silencing of *TaG6PDH2* during the wheat–*Pst* interaction by the BSMV-VIGS system. (**A**) Viral symptoms of BSMV-inoculated wheat leaves at 13 dpi. Mild chlorotic mosaic phenotypes were found in wheat leaves inoculated with BSMV: *TaG6PDH2-as1/2.* BSMV: *TaPDS*-inoculated wheat leaves acted as a positive control. Wheat leaves inoculated with FES buffer and BSMV: *γ* were used as the negative control. (**B**) Disease symptoms of the BSMV-pretreated fourth leaves inoculated with *Pst* CYR23 at 12 dpi. Spores, indicated by arrows. (**C**,**D**) Relative transcript profiles of *TaG6PDH2*, *TaPR1*, *TaPR2*, and *TaPR5* in *TaG6PDH2-*silenced wheat compared with negative control. The *Pst*-CYR23-infected wheat leaves were sampled at 0, 24, 48, and 120 hpi. Ta*EF* was used as an internal control. Values indicate means ± SDs from three independent samples. Asterisks represent significant differences (*p* < 0.05) according to Student’s *t*-test.

It was deemed that G6PDHs are involved in the ROS burst in plant defense responses, limiting the colonization and infection of pathogens [16,17]. We used a VIGS system to investigate whether *TaG6PDH2* contributes to wheat resistance to *Pst*. Two specific fragments (*TaG6PDH2*-1/2as) were chosen to knock down the expression of *TaG6PDH2* in wheat. Mild chlorotic mosaic symptoms were found in the BSMV-challenged wheat seedlings at 9 days post-inoculation (dpi), while no apparent growth defects were observed. Moreover, an evident photobleach phenotype was observed at 13 dpi in the *TaPDS*-silenced wheat seedlings (Figure 4A), demonstrating that the VIGS system operated successfully. The fourth leaves of the BSMV-challenged wheat seedlings were inoculated with CYR23 and displayed HR symptoms at 12 dpi. Some spores were found in the BSMV:*TaG6PDH2*-inoculated wheat plants compared to the BSMV:*γ* plants (Figure 4B). The silencing efficiency of *TaG6PDH2* in BSMV:*TaG6PDH2*-inoculated wheat leaves was analyzed by qRT-PCR. The transcript abundance of *TaG6PDH2* was substantially reduced in *TaG6PDH2*-silenced wheat seedlings compared with the controls at 0, 24, 48, and 120 hpi (Figure 4C), revealing that the expression of *TaG6PDH2* was efficiently silenced by the VIGS system. We also used qRT-PCR to measure the expression of three PR genes, *TaPR1*, *TaPR2*, and *TaPR5,* and found that the transcript levels of all three were notably suppressed in *TaG6PDH2*-silenced wheat plants (Figure 4D), indicating that a knockdown of *TaG6PDH2* may attenuate wheat resistance to *Pst*. As G6PDHs are involved in regulating redox homeostasis, we next assayed the transcript levels of two ROS-metabolism-correlated antioxidant enzyme genes, *TaSOD* and *TaAPX*. The expressions of both genes were decreased in *TaG6PDH2*-knockdown wheat leaves compared to the controls (Supplementary Figure S6).

### 2.6. Knockdown of TaG6PDH2 Attenuated Host Response and Facilitated Fungal Growth

**Figure 5:**
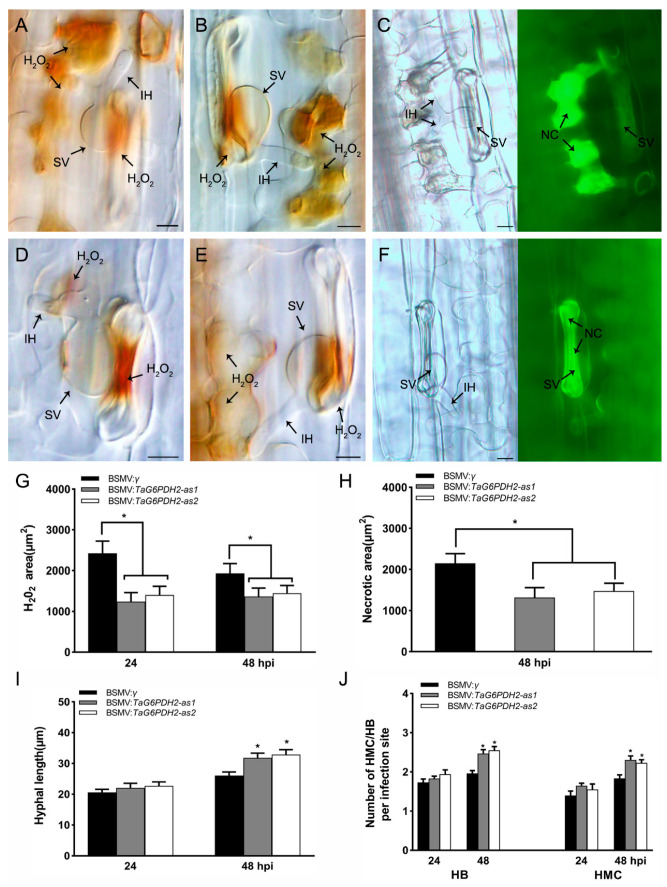
Histological observation of host response on the silenced wheat leaves infected with *Pst* CYR23. A-C. H_2_O_2_ accumulation, detected by DAB staining, at 24 hpi (**A**) or 48 hpi (**B**) and the necrotic cells at 48 hpi (**C**) in BSMV:*γ*-treated plants as a negative control. D-F. H_2_O_2_ accumulation at 24 hpi (**D**) or 48 hpi (**E**) and the necrotic cells at 48 hpi (**F**) in BSMV:*TaG6PDH2*-treated plants. Scale bars, 20 μm. SV, substomatal vesicle. IH, infection hypha. NC = necrotic cell. (**G**) H_2_O_2_ accumulation per infection site in the silenced wheat leaves, recorded at 24 and 48 hpi. (**H**) Necrotic area per infection site in the silenced wheat leaves, recorded at 48 hpi. (**I**) Hyphal length per infection site in the silenced wheat leaves, recorded at 24 and 48 hpi. (**J**) The numbers of haustorial mother cells (HMC) and branches of infection hyphae (HB) per infection site in the silenced wheat leaves, recorded at 24 and 48 hpi. At least 30 infection sites from randomly selected leaf segments were counted for each replicate. Each data point represents the mean ± SD from three independent biological repetitions. Asterisks represent significant differences (*p* < 0.05) compared with controls, according to Student’s *t*-test.

To investigate the involvement of *TaG6PDH2* in the host response, we detected H_2_O_2_ accumulation in *TaG6PDH2-*silenced wheat seedlings inoculated with the *Pst* avirulent pathotype CYR23. H_2_O_2_ accumulation was significantly reduced compared to the controls (Figure 5A,B,D,E,G). Furthermore, we found that the amount of necrotic area per infection site in *TaG6PDH2-*knockdown wheat leaves was much lower than in the controls (Figure 5C,F,H). These results indicate that silencing *TaG6PDH2* led to reduced HR and ROS accumulation during wheat–*Pst* interactions. To observe fungal growth in *TaG6PDH2-*silenced wheat seedlings, we performed histological observation of the mycelial structures of *Pst*. The numbers of haustorial mother cells (HMCs) and branches of infection hyphae (HB) as well as hyphal length (Figure 5I,J and Figure S7) increased relative to the controls at 48 hpi, whereas no significant differences were observed at 24 hpi. In conclusion, *TaG6PDH2* promotes wheat resistance against stripe rust by elevating ROS accumulation, which triggers cell death to prevent *Pst* invasion.

### 2.7. TaG6PDH2 Interacts with TaGrxS4

**Figure 6:**
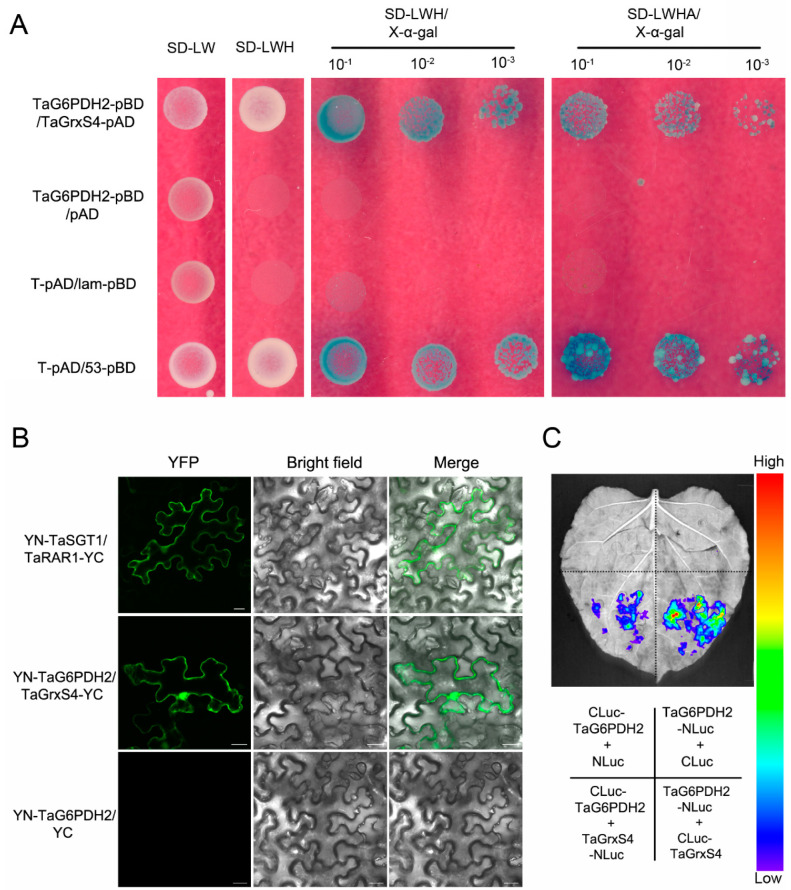
TaG6PDH2 interacts with TaGrxS4. (**A**) TaG6PDH2 interacts with TaGrxS4 in the Y2H system. Yeast strain AH109, containing the indicated pairs of plasmids, was normally grown on selective media SD/−LW, SD/−LWH, or SD/−LWHA containing 20 mg/mL X−α−gal. The growth conditions of the plates were observed and photographed 3 days after inoculation. SD, synthetic dropout. W, Tryptophan. L, Leucine. H, Histidine. A, Adenine. (**B**) BiFC analysis of the interaction of TaG6PDH2 and TaGrxS4. Co−infiltration of YN−TaSGT1 and TaRAR1−YC was used as a positive control (upper); Co−infiltration of YN-TaG6PDH2 and TaGrxS4−YC (middle); co-infiltration of YN−TaG6PDH2 and YC was used as a negative control (bottom). BF (bright-field) and YFP fluorescence (green) images were obtained using an Olympus FV3000 confocal microscope. Scale bars, 20 μm. (**C**) Luciferase complementation assay to verify the interaction of TaG6PDH2 and TaGrxS4. Co-expression of the indicated combinations mediated by agrobacterium in *N. benthamiana* leaves. All assays were repeated independently at least three times with comparable results.

To better understand the mechanism by which TaG6PDH2 participates in wheat resistance to *Pst*, we conducted yeast two-hybrid (Y2H) screening to identify potential interaction partners using TaG6PDH2 as bait. The TaG6PDH2 self-activation test revealed no basal expression of HIS3 in the Y2H system (Supplementary Figure S8). Several interesting candidate interactors for TaG6PDH2 were selected (Supplementary Table S1) for verification by co-expressing TaG6PDH2 and the potential targets on SD medium and staining with X-α-gal. Only TaG6PDH2 combined with wheat glutaredoxin, TaGrxS4, gave a positive signal in the Y2H system (Figure 6A). A sequence analysis showed that a 558 bp *TaGrxS4* encodes a protein consisting of 185 aa with a calculated molecular weight (Mw) of 19.95 kDa and a predicted pI of 6.82. A further analysis showed that typical conserved active sites (CGFS) were found in TaGrxS4, indicating that TaGrxS4 belongs to the CGFS type. 

To determine the interaction between TaG6PDH2 and TaGrxS4 in plants, we performed a bimolecular fluorescence complementation (BiFC) assay using transient expression in tobacco leaves. *Agrobacterium tumefaciens* containing the recombinant construct nYFP-G6PDH2 or cYFP-TaGrxS4 was co-infiltrated into *N. benthamiana* leaves. The combination of nYFP-TaSGT1 and cYFP-TaRAR1 was used as the positive control, and nYFP-G6PDH2 combined with cYFP was employed as the negative control. Strong fluorescence signals were detected in the positive control and the combination of nYFP-G6PDH2 and cYFP-TaGrxS4, whereas no fluorescence signal was observed in the negative control (Figure 6B). We then carried out a luciferase complementation (LUC) assay to further confirm the TaG6PDH2-TaGrxS4 interaction. strong luminescence signals were observed in regions of *N. benthamiana* leaves co-expressing *TaG6PDH2*-NLuc and CLuc-TaGrxS4, and TaGrxS4-NLuc and CLuc-TaG6PDH2. However, no luminescence signals were detected in negative controls co-expressing TaG6PDH2-NLuc and CLuc, or CLuc-TaG6PDH2 and NLuc (Figure 6C). These results, taken together, show that TaG6PDH2 physically interacts with TaGrxS4 in yeasts and plants.

## 3. Discussion

Although *G6PDHs* are known to serve as pivotal regulators in plant stress responses, few studies have addressed the functions of G6PDHs in wheat–*Pst* interactions. In the present study, a chloroplast-localized P2-type TaG6PDH2 from wheat was identified during *Pst* infection. The expression of *TaG6PDH2* was analyzed under different treatments. The heterologous expression of *TaG6PDH2* complemented resistance in the yeast mutant strain (*∆zwf1*) to oxidative stress. Silencing *TaG6PDH2* during the wheat–*Pst* interaction led to reduced ROS accumulation and compromised the host immune response. These results suggest that *TaG6PDH2* may enhance wheat resistance to stripe rust by elevating the NADPH supply for ROS production during *Pst* infection.

The subcellular location of proteins is essential for determining protein functions and understanding complex physiological processes [27]. G6PDHs mainly exist in the cytoplasm and chloroplasts of plants and exhibit distinct biological functions. In this study, TaG6PDH2 was classified into the P2-G6PDH type by a phylogenetic analysis, consistent with the subcellular localization prediction of TaG6PDH2. The temporal expression of *TaG6PDH2* in tobacco leaves and wheat protoplasts provided further support for determining its subcellular localization and indicated that TaG6PDH2 is localized to the chloroplasts. In addition, heterologous mutant complementation and prokaryotic expression revealed TaG6PDH2 to have G6PDH enzyme activity. These results suggest that *TaG6PDH2* encodes a chloroplast-localized G6PDH. Interestingly, in comparison with Arabidopsis, the G6PDH family members appear to be expanded in wheat (Supplementary Figure S1), suggesting that TaG6PDHs could possess many more functions during wheat growth and stress responses.

Numerous studies have found that the *G6PDH* gene in plants responds to biotic stress. For example, in Arabidopsis, the expression levels of all six G6PDH isoforms were increased upon root-knot nematode (RKN) infection, with the maximum induction for Cy-G6PDH *G6PD6* at 2 d after inoculation (dai) [28]. G6PDH activity was also induced in tobacco by infection with *Phytophthora nicotianae* [29]. In this study, *TaG6PDH2* was significantly induced in incompatible wheat–*Pst* interactions. Considering the lower sensitivity of P2-G6PDHs to NADPH inhibition [11], we infer that the P2-type TaG6PDH2 is favorable for NADPH oxidase-dependent ROS production and further HR cell death during wheat resistance to *Pst*. Furthermore, the transcript level of *TaG6PDH2* was also upregulated by hormone elicitors, especially SA and MeJA, which are known plant defense hormones [30,31]. Therefore, we assume that the transcriptional regulation of *TaG6PDH2* in *Pst*-infected wheat plants is associated with SA- and MeJA-related signaling pathways.

G6PDHs play a key role in plant immunity. For example, the Cy-type *G6PD6* contributes to Arabidopsis resistance against the bacterial pathogen *Pseudomonas syringae* [32]. Scharte and colleagues found that a P2-type *G6PDH* engineered for cytosolic expression confers enhanced tobacco resistance to pathogens [29]. In the current study, silencing *TaG6PDH2* through the VIGS system attenuated wheat resistance to *Pst*. Histologically, H_2_O_2_ accumulation and the size of the necrotic area were notably decreased. Accordingly, the expression of defense-related PR genes was downregulated in *TaG6PDH2*-silenced wheat seedlings. These results indicate that *TaG6PDH2* is responsible for wheat tolerance to stripe rust. A similar phenomenon has been observed in that the loss of function of two cytosolic isoforms of *G6PDHs* resulted in increased susceptibility of *Arabidopsis* to RKNs. Additionally, ROS production and defense response gene expression were suppressed in the mutant *Arabidopsis* infected with RKNs [28]. A possible model has been proposed to elucidate the functions of the P2-type G6PDHs:The NADPH produced by the P2-type G6PDHs in the chloroplasts is changed into NADH and translocated to the cytosol via the malate-oxaloacetate shuttle. Then, NADK1-mediated NADH phosphorylation renewably catalyzes the formation of NADPH, which is used as the substrate of RBOHs [29,33]. It is also worth noting that the ROS-metabolism-related genes *TaSOD* and *TaAPX* were downregulated in *TaG6PDH2*-silenced wheat plants, which is likely attributable to reduced ROS accumulation due to a decrease in NADPH (the substrate of NADPH oxidase) production caused by the knockdown of *TaG6PDH2*. These findings suggest that *TaG6PDH2*-mediated wheat resistance against *Pst* results from a contribution to ROS accumulation.

G6PDH activity is critical to plant stress tolerance and is tightly regulated. For example, the activity of a cytosolic G6PDH (G6PD6) was increased by phosphorylation through glycogen synthase kinase 3 (ASKα), which is necessary for maintaining the cellular redox homeostasis in Arabidopsis [34]. In addition, Nee et al. found that plastidic G6PDH activity is regulated by thioredoxins (TRXs) under light conditions, which are essential for the adaptation of oxidative PPP (OPPP) and efficient photosynthesis in plants [9]. It should be noted that two conserved cysteine residues of plastidic G6PDHs related to the reducible disulfide bridge and redox regulation were identified in potatoes [35]. In the present study, TaG6PDH2 was confirmed to interact with the wheat glutaredoxin TaGrxS4. Glutaredoxins, a small class of redox proteins in the thioredoxin superfamily, can regulate target protein activity by changing their redox (reduction/oxidation) state [36,37]. The alignment of several plant plastidic G6PDH polypeptide sequences showed that these two cysteine residues are conserved in plastidic G6PDHs, indicating that the redox regulation of plastidic G6PDH activity possibly proceeds via a similar mechanism. We thus speculate that TaGrxS4 is involved in the redox regulation of TaG6PDH2 activity, as stated above. Interestingly, we found that the interaction assay of TaG6PDH2 and TaGrxS4 by BiFC occurred in the cytoplasm rather than the chloroplast, which seems to indicate that the redox regulation of TaG6PDH2 by TaGrxS4 was carried out before TaG6PDH2 was translocated into the chloroplast. We speculate that TaGrxS4 may reductively inactivate TaG6PDH2 in the cytoplasm to avoid the competitive inhibition of Cy-G6PDH in wheat. Future work is needed to further clarify these hypotheses and underlying mechanisms. 

## 4. Materials and Methods

### 4.1. Experimental Materials, Culture Conditions, and Treatments 

Tobacco (*Nicotiana benthamiana)* and wheat (*Triticum aestivum* L.) seedlings were cultured in a greenhouse as previously described [38]. The inoculation with *Pst* and other treatments of wheat seedlings were performed as previously described [39]. The *Saccharomyces cerevisiae* mutant strain YNL241c was used in this study.

### 4.2. Cloning of TaG6PDH2 and Sequence Analysis 

The open reading frame (ORF) of *TaG6PDH2* was cloned from the *Pst*-infected wheat complementary DNA (cDNA). The specific primers (Supplementary Table S2) used for the PCR amplification of *TaG6PDH2* were designed based on the transcriptome sequencing of *Pst*-infected wheat leaves [23]. The obtained sequence was subsequently matched via BLAST against the *T. aestivum* cv. Chinese Spring (CS) genome, and related sequences and chromosomal locations were identified using Ensembl Plants (http://plants.ensembl.org) (accessed on 12 September 2020). Multiple sequence alignments were performed using the DNAMAN software (Lynnon Biosoft version 6.0). The sequences of G6PDHs from wheat and *Arabidopsis thaliana* were obtained from Ensembl Plants(accessed on 12 September 2020), and a phylogenetic tree was constructed using the MEGA software(Mega Limited version 6.0) through the neighbor-joining (NJ) method [40]. The Mw and theoretical pI of TaG6PDH2 were analyzed using the Compute pI/Mw tool (http://web.expasy.org/compute_pi/) (accessed on 12 September 2020). The subcellular localization of TaG6PDH2 was predicted using LOCALIZER (http://localizer.csiro.au/index.html) (accessed on 12 September 2020). Conserved domains were predicted using the Pfam database (http://pfam.xfam.org/)(accessed on 12 September 2020). All primers used in this study were designed with Primer Premiersoftware (Premier version 6.0) (Supplementary Table S2).

### 4.3. Subcellular Localization of TaG6PDH2

To clarify the subcellular localization of TaG6PDH2, the CDS of *TaG6PDH2* were inserted into two expression vectors, pCAMBIA1302 and pTF486, to generate the recombinant plasmids pCAMBIA1302-*TaG6PDH2* and pTF486-*TaG6PDH2*. Another recombinant plasmid, pCAMBIA1302-*TaG6PDH2∆cTP*, was constructed by deleting the TaG6PDH2 cTP. Subsequently, pCAMBIA1302-*TaG6PDH2* and pCAMBIA1302-*TaG6PDH2∆cTP* were individually transformed into the *A. tumefaciens* strain GV3101 and injected into 5-week-old tobacco leaves. To further verify the subcellular localization of TaG6PDH2, the construct pTF486-*TaG6PDH2* was introduced into wheat protoplasts. The preparation of the wheat protoplasts and the transformation of TaG6PDH2-GFP were conducted following a previously described protocol [41]. The empty vectors pCAMBIA1302 and pTF486 were used as controls. The fluorescence signals were detected using a confocal laser microscope (Olympus, Tokyo, Japan), and the assay was independently repeated at least three times.

### 4.4. RNA Isolation and Quantitative Real-Time PCR (qRT-PCR) Analysis

To measure the *TaG6PDH2* expression levels in the different treatments, wheat leaves were sampled at the denoted time points. RNA extraction and cDNA synthesis were performed as previously described [22]. The *TaG6PDH2* transcript levels were analyzed using qRT-PCR. Specific primers of the *TaEF-1α* gene (GenBank ID Q03033), an internal control for normalization, were designed and used in this assay. Representative qRT-PCR data from three biological repeats were further analyzed using the comparative 2^−△△Ct^ method [42].

### 4.5. Heterologous Expression of TaG6PDH2 in Yeast

To analyze the function of *TaG6PDH2*, we selected the *G6PDH*-deficient *S. cerevisiae* mutant strain YNL241c (*∆zwf1*). The CDS of *TaG6PDH2* was inserted into pDR195 to generate the recombinant plasmid pDR195-*TaG6PDH2*. The recombinant construct and the empty vector pDR195 were separately introduced into YNL241c, mediated by the lithium acetate (LiAc) method. The positive transformants carrying pDR195-*TaG6PDH2* or pDR195 (Supplementary Table S3) were cultured on a synthetic dropout (SD)-Ura medium. The yeast cells were subsequently washed and resuspended with sterilized water to an OD_600_ of 0.6. Then, 7 μL of a 10-fold serial dilution of cells was dropped on the SD-Ura medium containing different concentrations of H_2_O_2_. The yeast was cultured, and the cell growth condition was monitored. Three independent biological replicates were conducted for this assay.

### 4.6. Prokaryotic Expression and Enzymatic Characterization of TaG6PDH2

For the enzymatic characterization of TaG6PDH2, the CDS of *TaG6PDH2* was introduced into the vector pET15b-sumo to generate the recombinant plasmid pET15b-sumo-*TaG6PDH2,* which was then introduced into *Escherichia coli* BL21 competent cells. Next, the positive transformants were cultured in LB medium at 37 °C to an OD_600_ of 0.6, and fusion protein expression was induced using 0.1 mM IPTG overnight at 28 °C. The harvested cells were suspended in lysis buffer and lysed by sonication. The supernatant containing the soluble proteins was collected and further confirmed by a sodium dodecyl sulfate–polyacrylamide gel electrophoresis (SDS-PAGE) analysis using Coomassie brilliant blue (CBB) staining. The purification of the TaG6PDH2 fusion protein was conducted using a His-Trap FF affinity column (GE Healthcare, Uppsala, Sweden). The enzymatic characterization of TaG6PDH2 was subsequently conducted as previously described [43]. The double-reciprocal plots were used to determine the kinetic parameters of TaG6PDH2 by testing the TaG6PDH2 activity under varying substrate concentrations. This assay was repeated at least three times.

### 4.7. BSMV-Mediated TaG6PDH2 Gene Silencing

To investigate the possible function of *TaG6PDH2* in wheat–*Pst* interactions, the silencing of *TaG6PDH2* in wheat through the VIGS system was carried out following Chang et al. [44]. For silencing *TaG6PDH2* through VIGS, two specific sequence regions were selected from the cDNA sequence of *TaG6PDH2*. The recombinant plasmids were then constructed according to a previously described method [45]. Wheat seedling leaves were challenged with BSMV:*TaG6PDH2-as1* and BSMV:*TaG6PDH2-as2*. BSMV:*TaPDS*, wheat phytoene desaturase, and BSMV:*γ* were used as controls. The viral infection symptoms were observed and recorded at 13 dpi. Then, the leaves of BSMV-challenged wheat seedlings were further inoculated with the *Pst* pathotype CYR23. The disease phenotypes in BSMV-challenged wheat leaves were monitored and recorded at 12 dpi. *Pst*-wheat leaves were sampled at the indicated time points for further analysis. Three biological replicates were conducted.

To analyze the silencing efficiency of *TaG6PDH2*, the transcripts levels of *TaG6PDH2* in silenced plants were measured by qRT-PCR. In addition, the transcript levels of the pathogenesis-related (PR) genes *TaPR1* (GenBank ID AF384143), *TaPR2* (GenBank ID DQ090946), and *TaPR5* (GenBank ID FG618781) were analyzed [46]. The relative expression of the ROS-metabolism-related genes *TaAPX* (ascorbate peroxidase, GenBank ID JQ230568) and *TaSOD* (superoxidase, GenBank ID U69632) was measured using qRT-PCR in *TaG6PDH2*-silenced wheat [38]. 

### 4.8. Histological Observations of TaG6PDH2-Silenced Wheat Plants Infected with Pst

The host response and fungal growth were observed by microscopy in the *TaG6PDH2*-knockdown plants. H_2_O_2_ generated around the infection sites was specifically detected using 3,3-diaminobenzidine (DAB) [47]. The DAB staining, decolorization, and fixation of sampled leaves were conducted as previously described [48]. The H_2_O_2_ accumulation at infection sites in the leaves was monitored with an Olympus BX-51 microscope (Olympus, Tokyo, Japan) under a bright field and analyzed using cellSens Entry software (Olympus, Tokyo, Japan). Necrotic areas were also measured using a fluorescence microscope based on the auto-fluorescence of necrotic cells. To observe *Pst* infection structures, wheat leaf segments were stained with Alexa Fluor 488 (Invitrogen) conjugated wheat germ agglutinin (WGA) as previously described [49]. The numbers of hyphal branches (HB), haustorial mother cells (HMC), and haustorial hyphal lengths were measured using cellSens Entry software. Observations of no less than 30 infection sites from each randomly selected leaf segment were performed.

### 4.9. Y2H Assay

A Y2H screen with pGBKT7-*TaG6PDH2* was carried out as previously described [50]. The ORF of *TaG6PDH2* was cloned into the yeast vector pGBKT7 (Clontech, Tokyo, Japan). Total RNA isolated from *Pst*-infected wheat leaves was used for the construction of a pGADT7-cDNA library (Clontech, Tokyo, Japan). Potential yeast transformants containing cDNA clones interacting with TaG6PDH2 were selected on SD/-Leu/-Trp/-His (-LWH), SD/-Leu/-Trp/-His/-Ade (-LWHA), or SD/-LWHA/X-α-gal plates. Yeast transformation and further interaction assays were conducted according to the Yeast Protocols Manual (Clontech, Tokyo, Japan). pGBKT7-53 (murine p53) combined with pGADT7-T (SV40 large T-antigen) and pGBKT7-Lam (human lamin C) combined with pGADT7-T served as the positive and negative controls, respectively. The assay was independently performed at least three times.

### 4.10. Protein Interaction Assays in Plants

A BiFC assay was used to validate the interaction between TaG6PDH2 and TaGrxS4. The ORFs of *TaG6PDH2* and *TaGrxS4* were inserted into the binary vectors pSPYNE(R)173 and pSPYCE(M), respectively. For transient expression assays, *A. tumefaciens* strains containing pSPYNE(R)173-*TaG6PDH2* or pSPYCE(M)-*TaGrxS4* were co-injected into 5-week-old tobacco leaves. After two days, fluorescent signals and representative images were detected and photographed via FV3000 confocal microscopy with a 488 nm wavelength laser. 

To further verify the TaG6PDH2-TaGrxS4 interaction, an LUC assay was performed as previously described [51]. *TaG6PDH2* and *TaGrxS4* were recombined into pCAMBIA1300-NLuc (pNL) and pCAMBIA1300-CLuc (pCL), respectively. Transformation and co-infiltration were carried out as described above. After 48 h, the LUC images were captured immediately after spraying 1 mM luciferin onto the infiltrated leaves using a PlantView100 (BLT, Guangzhou, China) imaging apparatus. Three independent biological replicates were conducted for these assays.

## 5. Conclusions

In summary, this work sheds light on a positive role played by *TaG6PDH2* in host resistance to a rust pathogen, providing gene resources for breeding rust-resistant wheat cultivars. *TaG6PDH2* expression was induced during *Pst* infection, perhaps facilitating the PPP process and more NADPH production, which contributed to ROS production and ultimately led to cell death. Finally, HR in infection sites limited *Pst* colonization and hyphal spread. This finding elucidates the functions of G6PDHs in response to biotic stress in crop plants, helping to reveal the molecular mechanism of ROS burst in plant resistance against pathogen invasion.

## Data Availability

Not applicable.

## Supplementary Materials

The following supporting information can be downloaded at: https://www.mdpi.com/article/10.3390/ijms24010459/s1.
