# Supplementary material for: A Chloroplast-Localized Glucose-6-Phosphate Dehydrogenase Positively Regulates Stripe Rust Resistance in Wheat
Source: Int J Mol Sci. 2022 Dec 27;24(1):459. doi: 10.3390/ijms24010459 (PMC9820208; doi:10.3390/ijms24010459)
Supplement: Supplementary file 1 [file ijms-24-00459-s001.zip › ijms-2097729-supplementary.pdf]

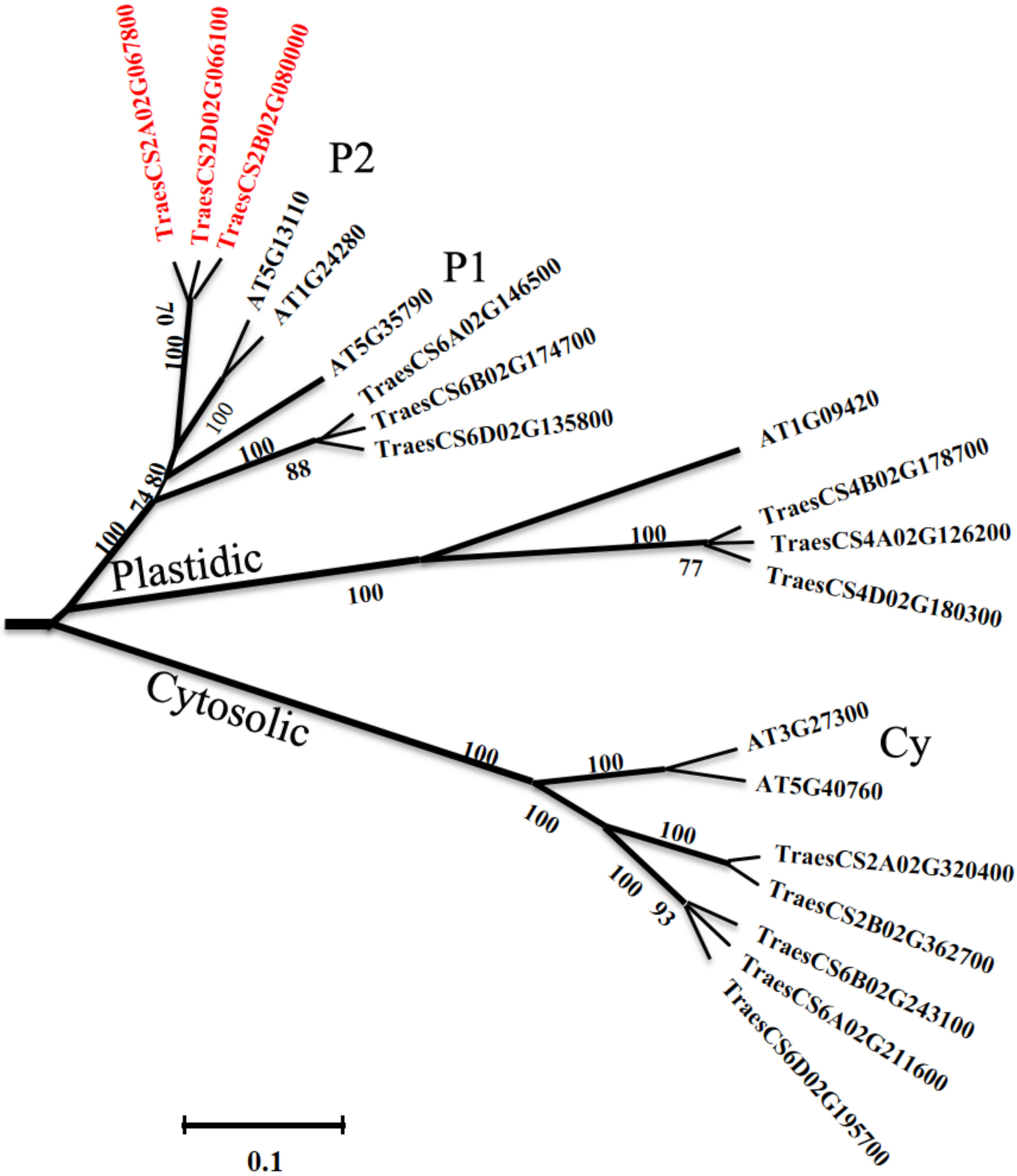

Supplementary Figure S1 Phylogenetic analysis of TaG6PDHs and AtG6PDHs. MEGA v6 software was used to conduct the analysis using the neighbor-joining method. 1,000 bootstrap replicates were used to calculate the confidence level of the groupings. The numbers adjacent to the branch points represent the percentage of replicates supporting each branch. Branches are labeled with the gene ID from the Ensembl Plants website (http://plants.ensembl.org) P1, AT5G35790 (G6PD1); P2, AT5G13110 (G6PD2) and AT1G24280 (G6PD3); Cy, AT3G27300 (G6PD5) and AT5G40760 (G6PD6).

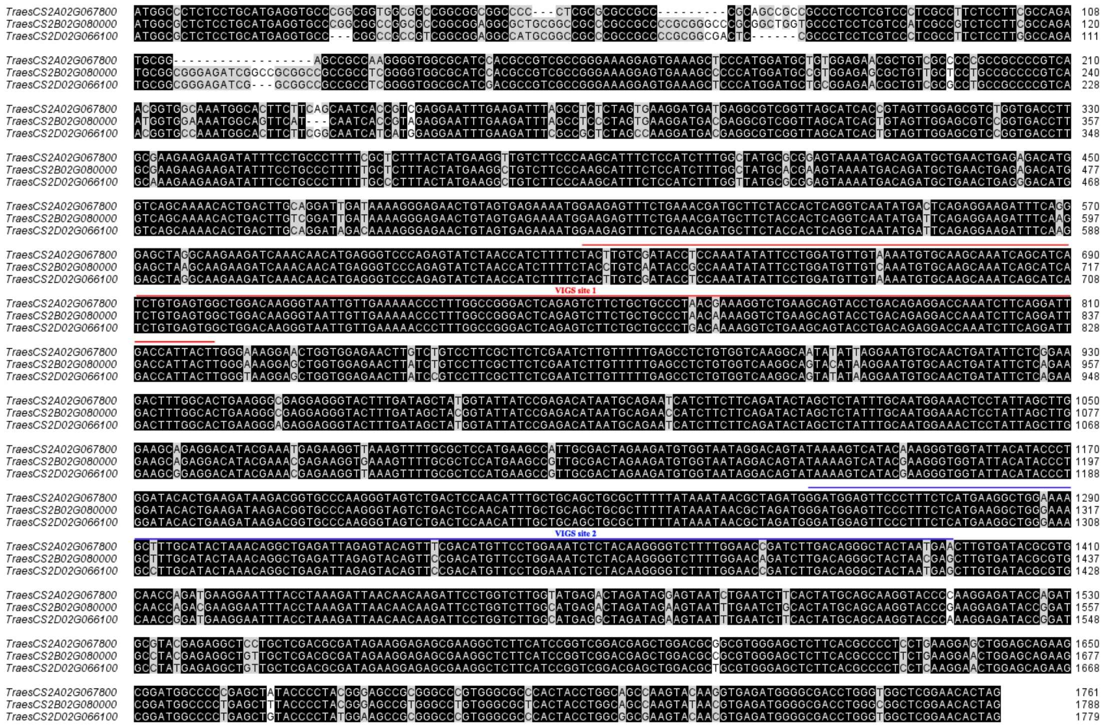

Supplementary Figure S2 Multi-alignment of the coding sequence of three copies of *TaG6PDH2*. The sequences were obtained from the wheat genome 2A, 2B, and 2D. Two specific regions for VIGS assay were indicated

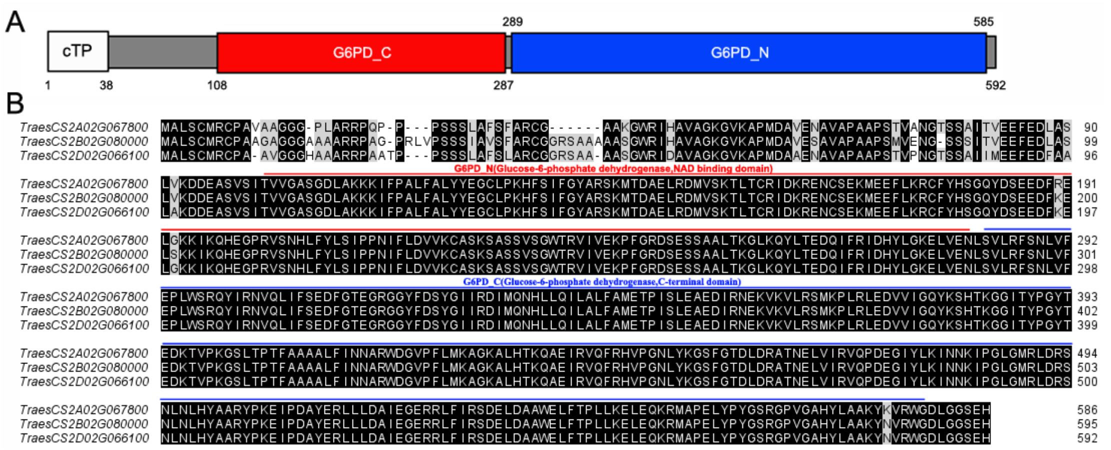

Supplementary Figure S3 Sequence analysis of TaG6PDH2. (A) Schematic diagram of the TaG6PDH2 domains. cTP, chloroplast-targeting sequence (G6PD_N = Glucose-6-phosphate dehydrogenase, NAD binding domain, G6PD_C = Glucose-6-phosphate dehydrogenase, C-terminal domain) (B) Multi-alignment of the TaG6PDH2 protein sequences. Sequences were obtained from the wheat genome 2A, 2B, and 2D. The domains of TaG6PDH2, G6PD_Ns, and G6PD_C are indicated.

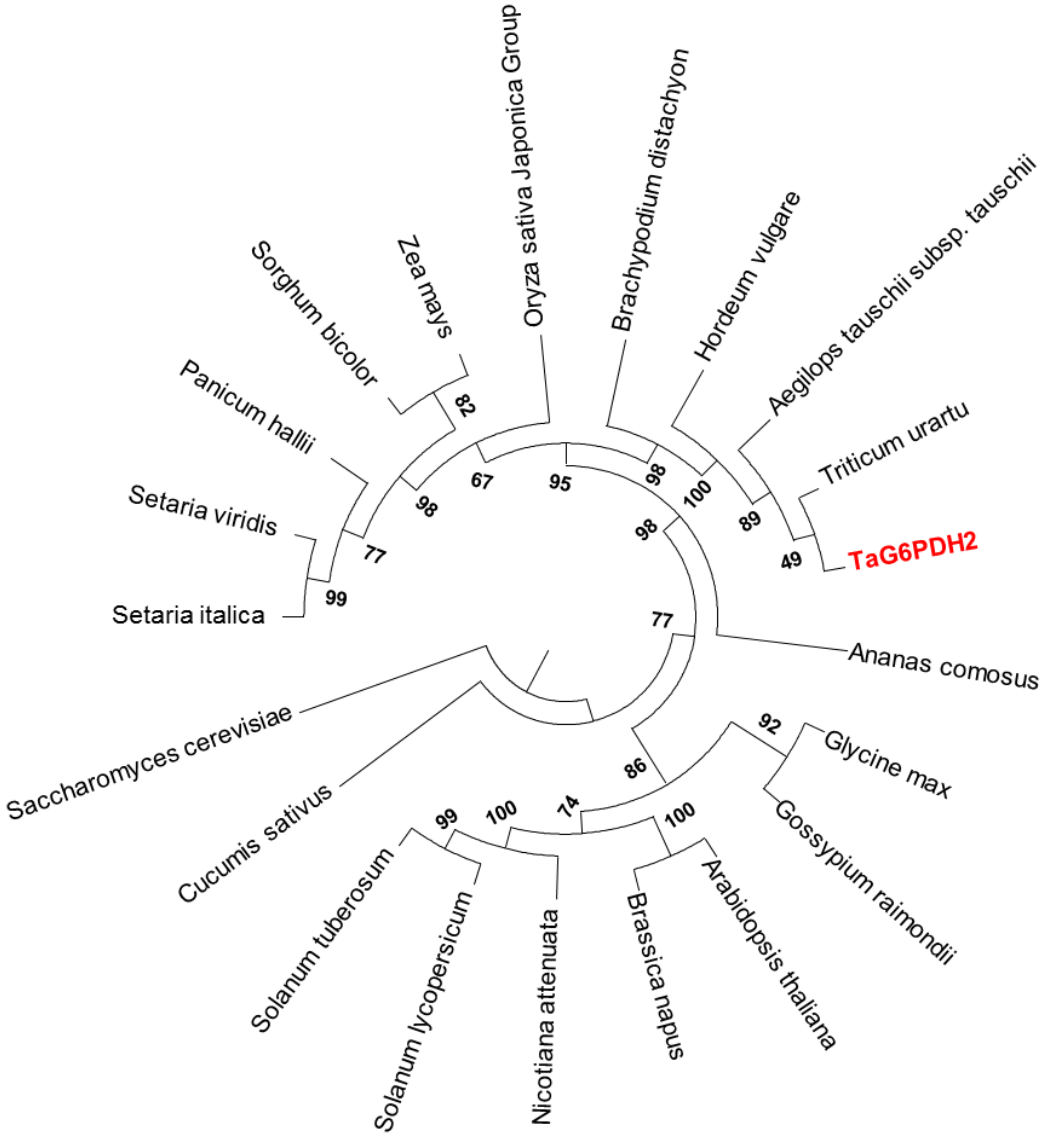

Supplementary Figure S4 Phylogenetic analysis of TaG6PDH2 and selected homologous proteins from other plants. MEGA v6 software was used to conduct the analysis using the neighbor-joining method. 1,000 bootstrap replicates were used to calculate the confidence level of the groupings. The numbers adjacent to the branch points represent the percentage of replicates supporting each branch. Branches are labeled with the species name.

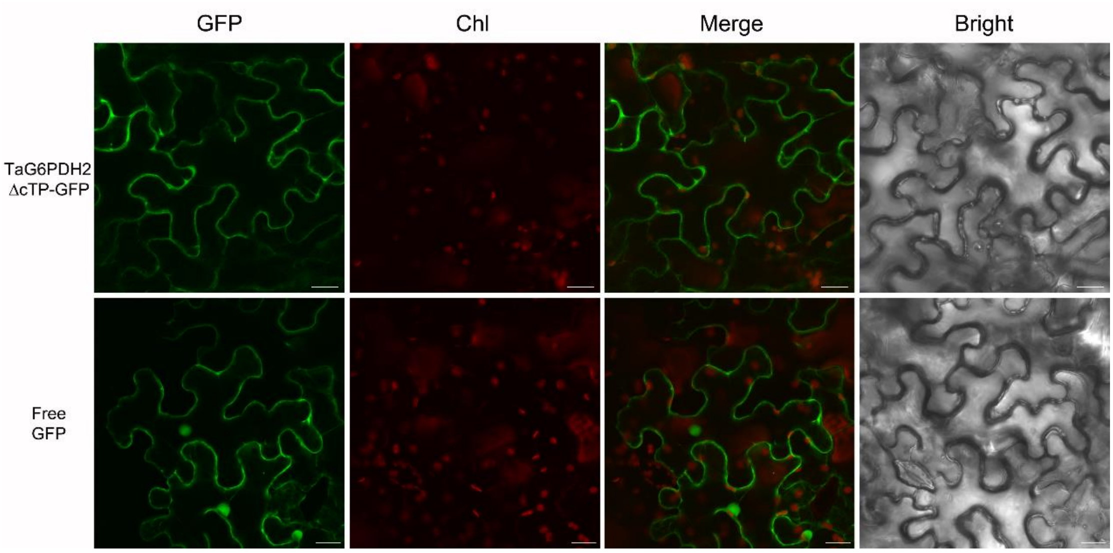

Supplementary Figure S5 Subcellular localization of TaG6PDH2ΔcTP in tobacco leaves. Transient expression of free GFP or TaG6PDH2ΔcTP-GFP fusion protein in *N. benthamianain* leaves mediated by *A.tumefaciens*. All signals were detected using an Olympus FV3000 confocal microscope. Scale bars, 20 μm. Chl, chlorophyll. GFP fluorescence is shown in green. Red fluorescence represents chlorophyll auto-fluorescence. Bright-field images indicate that identical fields were observed under white light. Merged GFP and chlorophyll images are presented.

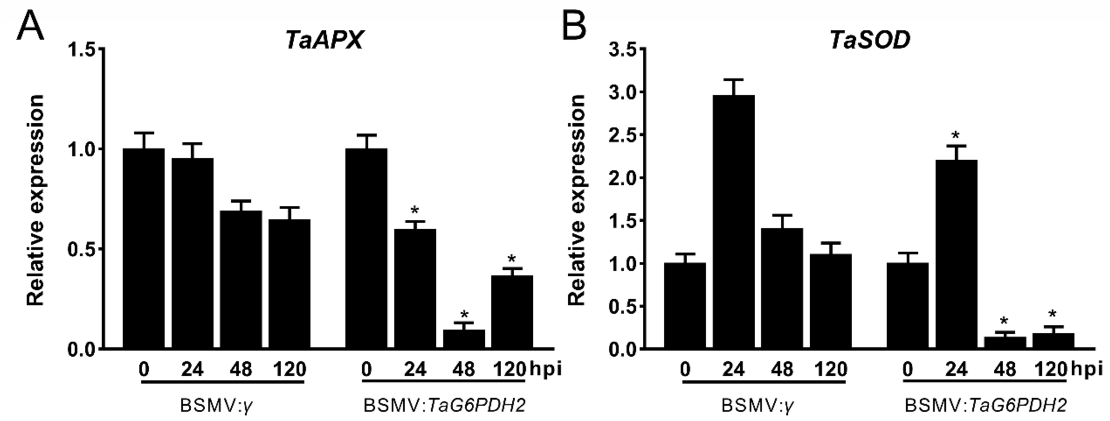

Supplementary Figure S6 Relative transcript levels of *TaAPX* (A) and *TaSOD* (B) in *TaG6PDH2*-knockdown wheat compared with negative control. *Pst* CYR23-infected wheat leaves were sampled at 0, 24, 48, and 120 hpi. Ta*EF* was used as an internal control. Values indicate mean ± SD from three independent samples. Asterisks represent significant differences ($P < 0.05$) according to Student's t-test.

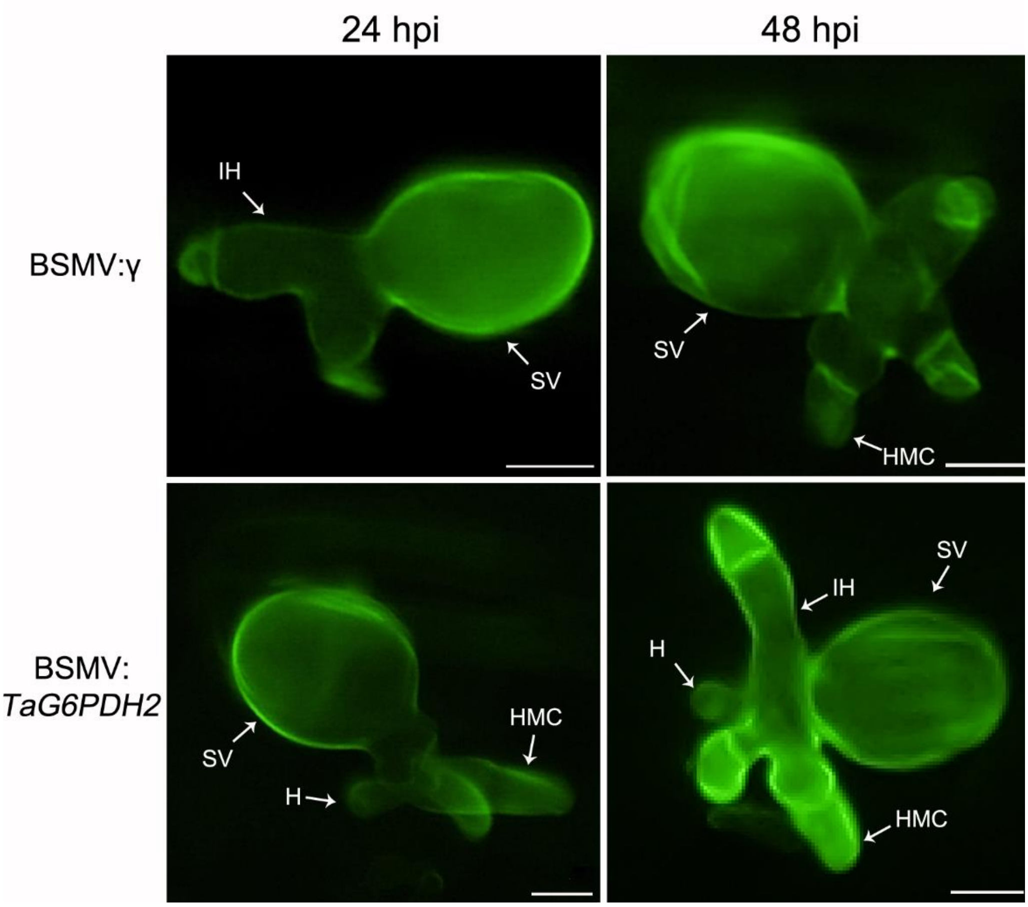

Supplementary Figure S7 Histological observation of host response on the silenced wheat leaves infected with *Pst* CYR23. Fungal growth, detected by WGA staining, at 24 hpi or 48 hpi in BSMV:*γ*- and BSMV:*TaG6PDH2*-treated plants. Scale bars, 20 μm. SV, substomatal vesicle. IH, infection hypha; HMC, haustorial mother cell; H, haustoria.

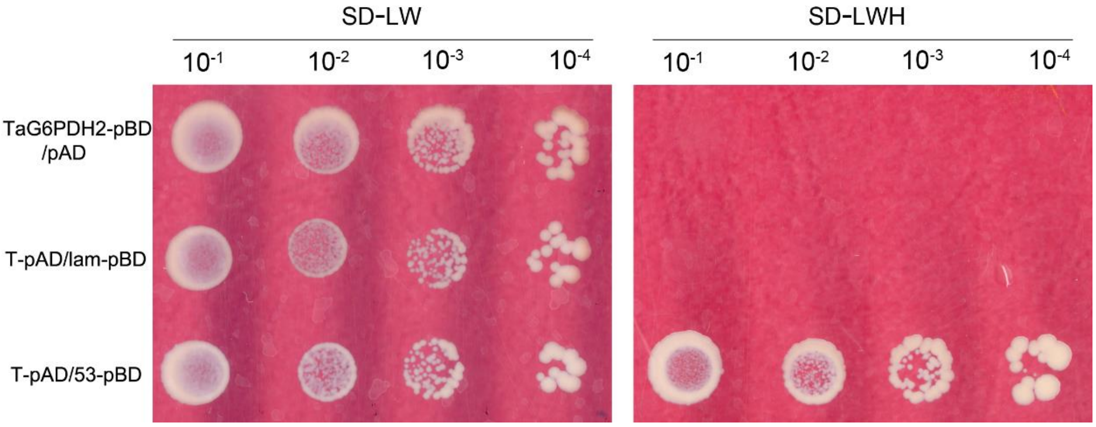

Supplementary Figure S8 Test for self-activation of *TaG6PDH2*. Yeast strain AH109 containing the indicated pairs of plasmids was normally grown on selective media SD/-LW or SD/-LWH. The growth condition of the plates was observed and photographed 3 days after inoculation. SD, synthetic dropout. W, Tryptophan. L, Leucine. H, Histidine.

Supplementary Table S1 Candidate interactors (CI)of TaG6PDH2 through the Y2H system

	Name	Genbank	Species	Times
CI1	70 kDa heat shock protein	GU452716	Triticum aestivum	6
CI2	monothiol glutaredoxin-S4	XM_044491837	Triticum aestivum	4
CI3	ToxA-binding protein 1	DQ235271	Triticum aestivum	4
CI4	ATP-dependent zinc metalloprotease FTSH 1	XM_020326324	Aegilops tauschii subsp. Tauschii	2
CI5	protoporphyrinogen oxidase	XM_020295601	Aegilops tauschii subsp. Tauschii	1

BLAST through NCBI. https://blast.ncbi.nlm.nih.gov/Blast.cgi

Supplementary Table S2 Primers used in this study.

Primers	Sequences (5′→3′)	
T-TaG6PDH2	F	ATGGCGCTCTCCTGCA
T-TaG6PDH2	R	CTAGTGTTCCGAGCCA
pTF486-TaG6PDH2	F	ACAGTCGACTCTAGAGGATCCATGGCGCTCTCCTGCA
pTF486-TaG6PDH2	R	GCCCTTGCTCACCATGGATCCGTGTTCCGAGCCA
pCAMBIA1302-TaG6PDH2	F	GGGGACTCTTGACCATGGGGATGGCGCTCTCCTGCA
pCAMBIA1302-TaG6PDH2ΔcTP	F	GGGGACTCTTGACCATGGGGATGGGCGGGAGATCG
pCAMBIA1302-TaG6PDH2	R	CTAGTCAGATCTACCATGGCGTGTTCCGAGCCA
Q-TaG6PDH2	F	TCTGAATAAGTGTGAGCGGTT
Q-TaG6PDH2	R	AGTTCCAATACAGCAGCCCA
Q-TaEF	F	TGGTGTCATCAAGCCTGGTATGGT
Q-TaEF	R	ACTCATGGTGCATCTCAACGGACT
pPDR195-TaG6PDH2	F	CAGCCTCGAGGGATCCATGGCGCTCTCCTGCA
pPDR195-TaG6PDH2	R	GTCCAAAGCTGGATCCCTAGTGTTCCGAGCCGCCCA
pET15b-TaG6PDH2	F	CAGATTGGCGGCGAATTCATGGCGCTCTCCTGCA
pET15b-TaG6PDH2	R	AGCCGGTTCCTCGAGGTGTTCCGAGCCA
VIGS-TaG6PDH2-as1	F	GCTAGCTGATTAATTAATACTTGTCGATACCTCCA
VIGS-TaG6PDH2-as1	R	GCTAGCTGAGCGGCCGCCAAGTAATGGTCAATCCTG
VIGS-TaG6PDH2-as2	F	GCTAGCTGATTAATTAAGGATGGAGTTCCCTTTCT
VIGS-TaG6PDH2-as2	R	GCTAGCTGAGCGGCCGCGCTCATTAGTAGCCCTGTCA
Q-TaPR1	F	AAACAACATCCATCAAAACCA
Q-TaPR1	R	ATTACCAGGTGGATCATAGTTACA
Q-TaPR2	F	AGGATGTTGCTTCCATGTTTGCCG
Q-TaPR2	R	AAGTAGATGCGCATGCCGTTGATG
Q-TaPR5	F	CAAGCAGTGGTATCAACGCAGAG
Q-TaPR5	R	GTGAAGCCACAGTTGTTCTTGATGTT
Q-TaSOD	F	TTGCCAATGCTGAGGGTG
Q-TaSOD	R	AATCCAGGTAAAACGAGAATG
Q-TaAPX	F	TGCGGATTTGTTCCAGTTGG
Q-TaAPX	R	GGCATGACCTTCCAAGTGTGTG
pGBKT7-TaG6PDH2	F	GAGGACCTGCATATGATGGCGCTCTCCTGCA
pGBKT7-TaG6PDH2	R	GATCCCCGGGAATTCCTAGTGTTCCGAGCCGCCCA
pGADT7-CI1	F	GGAGGCCAGTGAATTCATGGCTACCTTCACTTCCCA
pGADT7-CI1	R	CACCCGGGTGGAATTCTTAATTGCTATCTGTGAAG
pGADT7-CI2	F	GGAGGCCAGTGAATTCATGGCGAGGCTGGTGTC
pGADT7-CI2	R	CACCCGGGTGGAATTCTCACTGGGCACTGTCTTG
pGADT7-CI3	F	GGAGGCCAGTGAATTCATGGCGGCCATATCGTCG
pGADT7-CI3	R	CACCCGGGTGGAATTCTTAATGCTTCAAGGGATA
pGADT7-CI4	F	GGAGGCCAGTGAATTCATGGCGCCCCCTTCCCTCT

pGADT7-CI4	R	CACCCGGGTGGAATTCTTATGCGACGAACAGCTCT
pGADT7-CI5	F	GGAGGCCAGTGAATTCATGGTCGGCGCAACCATG
pGADT7-CI5	R	CACCCGGGTGGAATTCTCACTTGTAGGCATACTT
YNE-TaG6PDH2	F	GCCTACTAGTGGATCCATGGCGCTCTCCTGCA
YNE-TaG6PDH2	R	CGAGGTCGACGGATCCCTAGTGTTCCGAGCCGCCCA
YCE-TaGrxS4	F	CGCCACTAGTGGATCCATGGCGAGGCTGGTGTC
YCE-TaGrxS4	R	TACTATCGATGGATCCTCACTGGGCACTGTCTTG
pCAMBIA 1300-LUC-TaG6PDH2	F	ACGGGGGACGAGCTCGGTACCATGGCGCTCTCCTGCA
pCAMBIA 1300-LUC-TaG6PDH2	R	CGCGTACGAGATCTGGTCGACGTGTTCCGAGCCGCCCA
pCAMBIA 1300-LUC-TaGrxS4	F	ACGGGGGACGAGCTCGGTACCATGGCGAGGCTGGTGTC
pCAMBIA 1300-LUC-TaGrxS4	R	CGCGTACGAGATCTGGTCGACTCACTGGGCACTGTCTTG

Supplementary Table S3 *Saccharomyces cerevisiae* mutant strains and complementation strains used in this study.

Strains	Genotype/comment
Δ*zwf1*	YNL241c mutant
Δ*zwf1*+empty	Δ*zwf1* complemented with pDR195 vector
Δ*zwf1+TaG6PDH2*	Δ*zwf1* complemented with pDR195-*TaG6PDH2* vector

YNL241c (glucose-6-phosphate dehydrogenase, MET19, POS10, ZWF1)
